# Inhibition of PI3 kinase isoform p110α suppresses neuroblastoma growth and induces the reduction of Anaplastic Lymphoma Kinase

**DOI:** 10.1186/s13578-022-00946-9

**Published:** 2022-12-30

**Authors:** Yue Guo, Donghao Guo, Shaoting Zhang, Yuan Zhang, Xiaoyan He, Xiaohua Jiang, Andrew Man-Lok Chan, Lin Zou, Jianmin Sun, Hui Zhao

**Affiliations:** 1 Key Laboratory for Regenerative Medicine, Ministry of Education, School of Biomedical Sciences, Faculty of Medicine, The Chinese University of Hong Kong, Hong Kong SAR, China; 2 NHC Key Laboratory of Metabolic Cardiovascular Diseases Research, Science and Technology Center, School of Basic Medical Sciences, Ningxia Medical University, No. 1160 Shengli Street, Yinchuan, 750004 China; 3 Newborn Screening Center & Center for Clinical Molecular Medicine of Children’s Hospital, Chongqing Medical University, Chongqing, 400014 China; 4 Clinical Research Unit, Institute of Pediatric Infection, Immunity, and Critical Care Medicine, Shanghai Children’s Hospital, Shanghai Jiao Tong University School of Medicine, Shanghai, 200062 China; 5 Kunming Institute of Zoology Chinese Academy of Sciences - The Chinese University of Hong Kong Joint Laboratory of Bioresources and Molecular Research of Common Diseases, Hong Kong, SAR China; 6 Hong Kong Branch of CAS Center for Excellence in Animal Evolution and Genetics, The Chinese University of Hong Kong, Hong Kong, SAR China

**Keywords:** Neuroblastoma, PI3 Kinase, p110α, PIK3CA, ALK, Alpelisib

## Abstract

**Background:**

In neuroblastoma, hyperactivation of the PI3K signaling pathway has been correlated with aggressive neuroblastomas, suggesting PI3Ks as promising targets for the treatment of neuroblastoma. However, the oncogenic roles of individual PI3K isoforms in neuroblastoma remain elusive.

**Results:**

We found that PI3K isoform p110α was expressed at higher levels in neuroblastoma tissues compared with normal tissues, and its high expression was correlated with an unfavorable prognosis of neuroblastoma. Accordingly, PI3K activation in neuroblastoma cells was predominantly mediated by p110α but not by p110β or p110δ. Suppression of p110α inhibited the growth of neuroblastoma cells both in vitro and in vivo, suggesting a crucial role of p110α in the tumorigenesis of neuroblastoma. Mechanistically, inhibition of p110α decreased anaplastic lymphoma kinase (ALK) in neuroblastoma cells by decreasing its protein stability.

**Conclusions:**

In this study, we investigated the oncogenic roles of PI3K isoforms in neuroblastoma. Our data shed light on PI3K isoform p110α in the tumorigenesis of neuroblastoma, and strongly suggest the p110α inhibitors as potential drugs in treating neuroblastoma.

**Supplementary Information:**

The online version contains supplementary material available at 10.1186/s13578-022-00946-9.

## Background

Neuroblastoma is a neural crest-derived embryonic malignancy with high heterogeneity, causing 15% of cancer-related deaths in children [1]. Although the tumor can spontaneously regress in a group of young patients (< 18 months), half of the neuroblastoma patients are diagnosed with high-risk neoplasms, and around 50% of patients with high-risk neuroblastoma are not sensitive to initial treatment. Moreover, tumor relapse can happen in half of the high-risk neuroblastoma patients after the primary therapies [2]. Recently, molecular characterization of neuroblastoma has been advanced for precision prognosis and therapy stratification. Molecular targeted therapy becomes an important therapeutic approach to treat high-risk neuroblastoma, especially the relapsed and refractory neuroblastomas, which can hardly be cured by surgical resections and chemotherapies [2, 3].

N-MYC proto-oncogene protein (MYCN) and Anaplastic Lymphoma Kinase (ALK) are the two important oncogenic drivers of neuroblastoma. Overexpression of MYCN or the constitutively active mutant of ALK, e.g., ALK^F1174L^, in neural crest cells can induce neuroblastoma in mice [4, 5]. In the past decades, inhibitors suppressing the activities of ALK or MYCN have made some achievements in both preclinical and clinical models. However, drug resistance against ALK inhibitors and the absence of direct antagonists for MYCN limit the treatment of neuroblastoma in clinic [6–8].

PI3K-AKT-mTOR signaling pathway regulates key cellular processes, including cell survival, proliferation, motility, and genomic instability in cancer cells. Its dysregulation is one of the most frequent events in cancers [9]. PI3Ks are categorized into three classes based on their structures and substrates. The class IA PI3Ks are the most studied among all classes of PI3Ks. They are heterodimers, consisting of a regulatory subunit (p85a and its splicing variants p55α and p50α, p85β, or p55γ), and a catalytic subunit (p110α, p110β, or p110δ) [10, 11]. In neuroblastoma, over-activation of the PI3K signaling pathway has been reported in both tumor samples and cancer cell lines. However, unlike most PI3K signaling-driven tumors, mutations in *PIK3CA* and *PTEN* are infrequent in neuroblastoma, suggesting the distinct oncogenic mechanism of the PI3K signaling in the carcinogenesis of the neuroblastoma [12–15]. Several inhibitors targeting the PI3K-AKT-mTOR axis have been tested preclinically in neuroblastoma, but most of them failed for further clinical development due to the low therapeutic index [16–20].

In this study, we found that high expression of p110α was correlated with worse outcomes of neuroblastoma prognosis. Two p110α-targeted inhibitors, copanlisib and alpelisib, currently used in the clinic, showed high efficacy in our neuroblastoma models. Furthermore, we found that inhibition of p110α induced ALK instability and synergized the inhibitory effects of ALK inhibitors on neuroblastoma cells. Our results provide strong evidence to support the therapeutic application of p110α targeted inhibitors in treating neuroblastoma.

## Results

### High expression of p110α is correlated with worse treatment outcomes of neuroblastoma patients

**Fig. 1:**
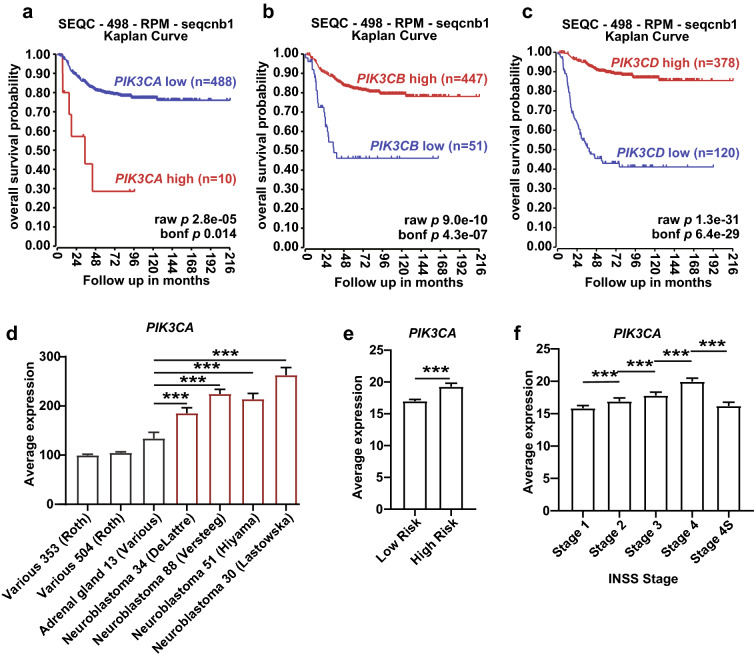
The expression of p110α is correlated with neuroblastoma prognosis. **a–c** Kaplan–Meier analysis of overall survival probability in neuroblastoma patients, separated by *PIK3CA* (**a**), *PIK3CB* (**b**), *PIK3CD* (**c**) expression. Cut-off values were selected by Kaplan Scan (“Materials and methods” section). SEQC (seqcnb1) dataset was analyzed. **d** Expression of *PIK3CA* in 3 normal tissue datasets and 4 neuroblastoma datasets. The bar chart is presented by mean ± SD. **e**–**f **Expression of *PIK3CA* in neuroblastoma samples. Patient samples were categorized by risk stratification (**e**) or neuroblastoma stages (**f**). The bar charts were presented as mean ± SEM. SEQC (seqcnb1) dataset was used for the analysis. All the datasets were obtained from the R2: Genomics Analysis and Visualization Platform (https://r2.amc.nl). The Student’s *t*-test was used for statistical analysis. ***p < 0.001

To gain insight into the function of class IA PI3K isoforms in neuroblastoma, we analyzed the gene expression data from primary neuroblastoma samples via the R2: Genomics Analysis and Visualization Platform (http://r2.amc.nl). We first compared the correlations between the different catalytic class IA PI3K isoforms and the neuroblastoma prognosis. The Kaplan–Meier analysis showed that high expression of *PIK3CA* was associated with poor overall survival in patients (Fig. 1a), while high *PIK3CB* or *PIK3CD* levels indicated more favorable overall survival probability (Fig. 1b, c). Similarly, event-free survival probability is also lower in the *PIK3CA* high, *PIK3CB* low, and *PIK3CD* low groups in the Kaplan–Meier analysis (Additional file 1: Fig. S1a–c). *PIK3CA,* indeed, was expressed at higher levels in neuroblastoma tissues than in normal tissues, including the adrenal gland (Fig. 1d). Moreover, *PIK3CA* expression is higher in the high-risk group than in the low-risk group (Fig. 1e). In the International Neuroblastoma Staging System (INSS)-stage stratified neuroblastoma samples, we found that higher *PIK3CA* expression was highly correlated to the more advanced stages (Fig. 1f).

### PI3K activation in neuroblastoma cells is predominantly mediated by p110α but not by p110β or p110δ

**Fig. 2:**
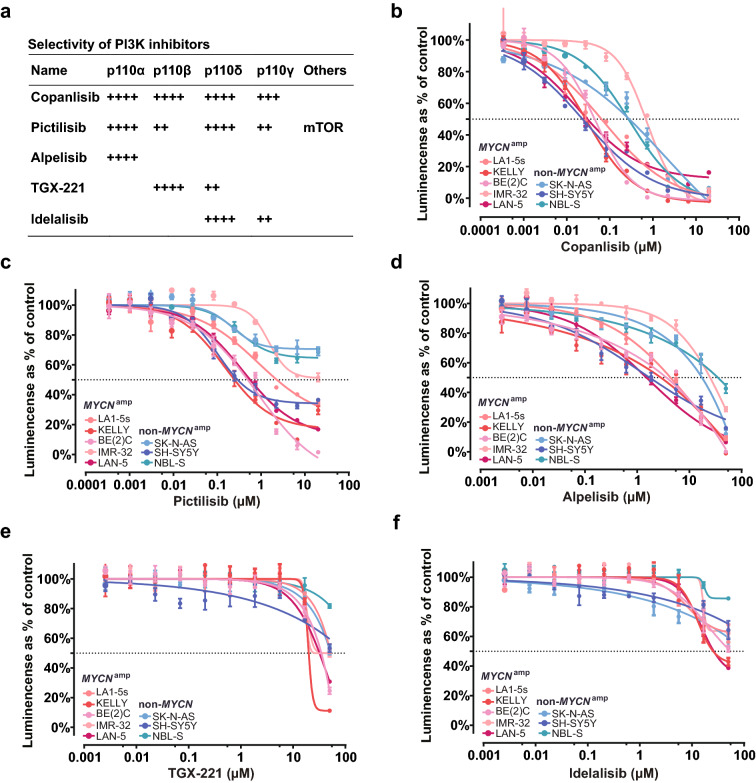
p110α inhibitors decrease the viability of neuroblastoma cells. **a** The specificity of different PI3K inhibitors. Information was collected from Selleckchem (https://www.selleckchem.com/PI3K.html). **b–f** Cell viabilities of different neuroblastoma cells were determined by CellTiter-Glo® Luminescent Cell Viability Assay upon treating with various PI3K inhibitors at different doses for 72 h. 10 μg/ml puromycin was applied for no-survival controls. All values were normalized to the vehicle (maxi) and no-survival control (min) before illustrating the inhibition curves with GraphPad Prism (“Materials and methods” section). Data were presented as the mean ± SD

In addition to the differential correlation of class IA PI3K isoforms with neuroblastoma patient outcomes, we tried to determine the isoform dependence of neuroblastoma to PI3Ks. The efficacy of PI3K inhibitors with distinct isoform specificity was compared in neuroblastoma cells (Fig. 2a). Considering the importance of MYCN and ALK during neuroblastoma tumorigenesis, cell lines with specific genetic alterations of *MYCN* and *ALK* have been included (Additional file 1: Fig. S2a). Five *MYCN* amplified cell lines (LA1-5S, IMR-32, BE(2)C, LAN-5, and KELLY) and three non-*MYCN* amplified cell lines (SH-SY5Y, NBL-S, and SK-N-AS) were selected to examine the cytotoxicity of different class IA PI3K inhibitors. Among these cell lines, SH-SY5Y and KELLY harbor *ALK*^*F1174L*^ mutation, while LAN-5 carries *ALK*^*R1275Q*^ mutant, and LA1-5s cells do not express ALK. The endogenous expression of *ALK* and *MYCN* in these cell lines was also evaluated in our assay to confirm the different levels of the two genes in the selected neuroblastoma cell lines (Additional file 1: Fig. S2b, c). *MYCN* is highly expressed in LAN-5, KELLY, BE(2)C, and IMR-32, while ALK is highly expressed in LAN-5, IMR-32, KELLY, NBL-S, and SH-SY5Y cell lines.

We found that copanlisib effectively inhibited cell viability of all the tested neuroblastoma cell lines (Fig. 2b). With IC50 values less than 1 µM in the cell lines, it is the most effective among the three p110α inhibitors in treating neuroblastoma cells (Additional file 1: Fig. S3). Another pan-PI3K inhibitor, pictilisib, was also cytotoxic to these neuroblastoma cell lines but less effective than copanlisib, as revealed by the inhibitory curve (Fig. 2c), despite that pictilisib treated NBL-S, IMR-32, and LA1-5s failed to reach 50% of inhibition due to its poor solubility (data not shown). Notably, cell viability of tested neuroblastoma cells could be reduced by less than 5 μM alpelisib (p110α-selective inhibitor) treatment (Fig. 2d). However, blocking p110β with TGX-221 or inhibiting p110δ with idelalisib could not affect cell proliferation of all tested neuroblastoma cell lines until the treatment concentration reached 10 μM (Fig. 2e, f), suggesting the reliance of neuroblastoma cell growth on p110α.

**Fig. 3:**
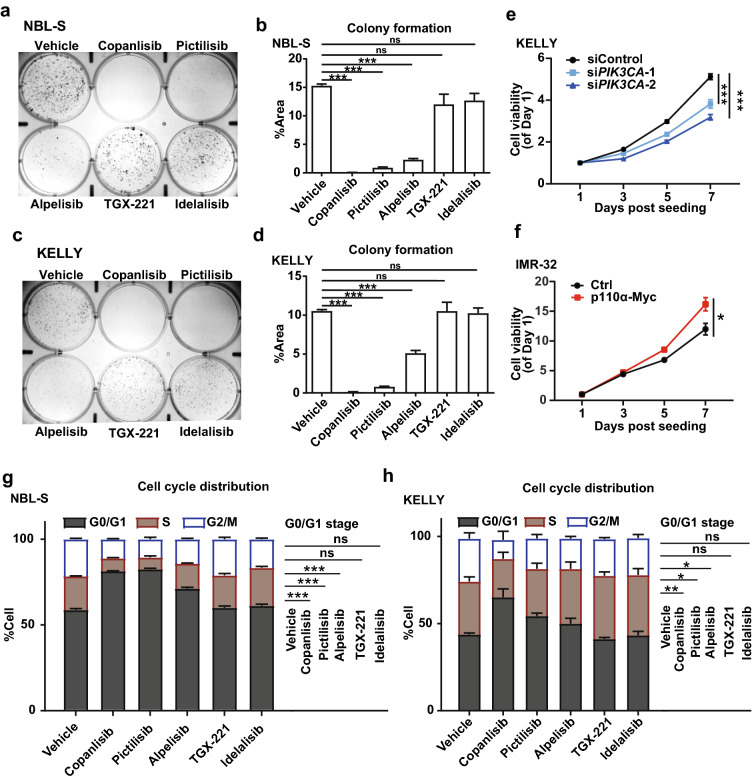
p110α inhibitors decrease cell proliferation of neuroblastoma cells. **a–d** Colony formation analysis of NBL-S cells (**a**, **b**) and KELLY cells (**c**, **d**) upon the treatment of different PI3K inhibitors (1 µM). Pictures were captured after 12-day treatment and afterwards crystal violet staining (**a**, **c**). Quantifications of all pictures were conducted by Image J (**b**, **d**). **e** Cell viability showing the cell growth after siRNA transfection. KELLY cells were seeded in 96-well plates at 6 h after si*PIK3CA* (50 nM) or siControl (50 nM) transfection. Cell viability was then determined by MTT assay at different time points. Data were expressed as mean ± SEM. **f** Cell viability showing the cell growth after p110α overexpression in IMR-32 cells. Cells were transfected with vector control or p110α-Myc and re-seeded into 96-wells after 24 h. Cell viability was determined by MTT assay at different time points. Data were expressed as mean ± SEM. **g**, **h** cell cycle distribution of NBL-S cells and KELLY cells treated with PI3K inhibitors as indicated (1 µM). Neuroblastoma cells were fixed after 24-h treatment with the PI3K inhibitors. The DNA content was then determined by PI staining and flow cytometry analysis. Cell cycle distribution was defined with Flow Jo. Data from 3 repeats were summarized. Bar charts were presented as mean ± SEM. The Student’s *t*-test was used for the statistical analysis of the G_0_/G_1_ proportion between the inhibitor-treated groups and the vehicle group. *p < 0.05; **p < 0.01; ***p < 0.001; ns, not significant

To further validate the inhibitory impacts of these PI3K inhibitors on neuroblastoma cells, we performed colony formation assays. Consistent with the cell viability data above, the three inhibitors targeting p110α showed potent inhibitory effects on both NBL-S and KELLY cells in colony formation (Fig. 3a–d). Alpelisib was less effective than the two pan-inhibitors, but it still reduced the cell colonies by more than 50% in the two cell lines (Fig. 3a–d). However, isoform-selective inhibitors targeting either p110β or p110δ did not apparently suppress the colony formation of NBL-S and KELLY cells (Fig. 3a–d). In addition, attenuation of p110α by the siRNAs also suppressed the growth of KELLY cells (Fig. 3e). Consistently, p110α overexpression increased cell growth in IMR-32 cells as well (Fig. 3f), confirming the specificity of p110α inhibitor treatments.

To examine whether PI3K inhibitors modulate the cell cycle of neuroblastoma cells, the cell cycle distribution was assessed upon the treatment of different inhibitors. As we expected, p110α inhibitors significantly increase the G_0_/G_1_ phase arrest in neuroblastoma cells (Fig. 3g, h). Of note, compared with the control, over 20% increase in the G_0_/G_1_ phase was induced by copanlisib treatment in NBL-S and KELLY cells (Fig. 3g, h and Additional file 1: Fig. S4b, h). The treatment of pictilisib also caused G_0_/G_1_ phase arrest in NBL-S and KELLY cells, increasing around 25% and 20% in G_0/_G_1_ proportion, respectively (Fig. 3g, h and Additional file 1: Fig. S4c, i). Similar to copanlisib and pictilisib, alpelisib caused more than 10% increment of G_0_/G_1_ phase cells (Fig. 3g, h and Additional file 1: Fig. S4d, j). Conversely, p110β or p110δ inhibitor could not affect the cell cycle in both tested cell lines (Fig. 3g, h and Additional file 1: Fig. S4e, f, k, l).

**Fig. 4:**
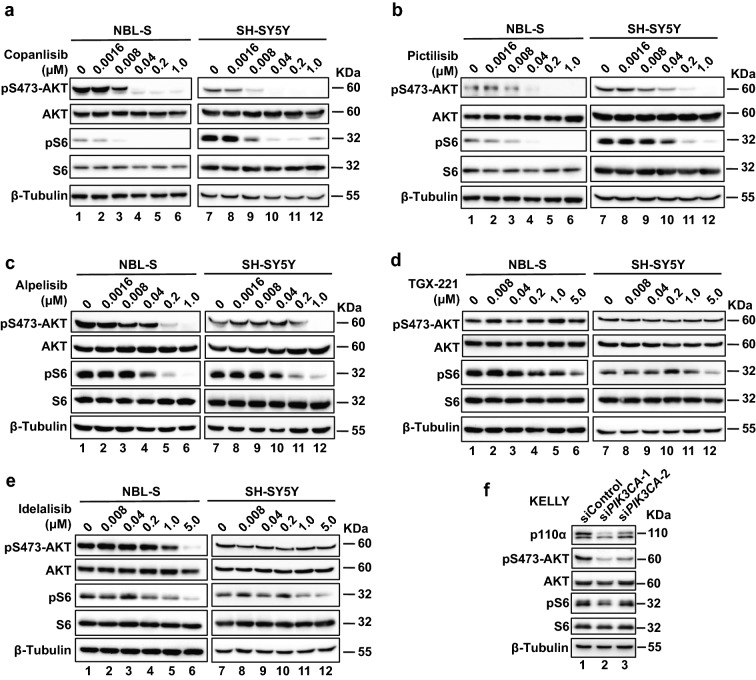
Inhibition of p110α suppresses the downstream effectors of the PI3K signaling pathway in neuroblastoma cells. **a–e** western blot showing inhibition of PI3K signaling in NBL-S cells or SH-SY5Y cells after PI3K inhibitors treatment (2 h). **f** PI3K signaling suppression revealed by western blot in KELLY cells, transfected with si*PIK3CA* (50 nM) or siControl (50 nM) for 48 h. β-Tubulin was blotted as the loading control

We next examined the activation of the PI3K signaling pathway under the treatment of different PI3K inhibitors. In agreement with the inhibition of neuroblastoma cell growth, the three p110α inhibitors suppressed the downstream signaling of PI3K in neuroblastoma cells (Fig. 4a–c). The activation of PI3K signaling could be largely blocked by 40 nM of copanlisib treatment in NBL-S and SH-SY5Y cells (Fig. 4a and Additional file 1: Fig. S5a, b). Pictilisib at 40 nM significantly affected the phosphorylation of both AKT and the downstream effector S6 (Fig. 4b and Additional file 1: Fig S5c, d). Alpelisib also evidently suppressed the phosphorylation of AKT and S6 at 0.2 μM (Fig. 4c and Additional file 1: Fig. S5 e, f), whereas no apparent reduction in the activation of AKT was detected upon the treatment of p110β inhibitor TGX-221 (Fig. 4d and Additional file 1: Fig. S5g, h). P110δ inhibitor idelalisib could barely inhibit the signaling pathway at concentrations less than 1.0 μM and failed to suppress the activation of the AKT at 5 μM treatment in SH-SY5Y cells (Fig. 4e and Additional file 1: Fig. S5i, j). Using siRNAs to inhibit p110α expression also downregulated the activation of AKT and S6 (Fig. 4f), suggesting the major role of p110α in PI3K signaling in neuroblastoma.

Although both pan-PI3K inhibitors (copanlisib and pictilisib) in our study were more effective than p110α selective inhibitor alpelisib in vitro, the inhibition on neuroblastoma cell growth by alpelisib was not further enhanced by either TGX-221 or idelalisib (Additional file 1: Fig. S6a, b), suggesting that p110α is the prevailing catalytic isoform in neuroblastoma cells among the class IA PI3Ks. This notion was further supported by examining the PI3K signaling pathway activation when the cells were treated by either alpelisib alone or combined with TGX-221 and idelalisib (Additional file 1: Fig. S6c).

### p110α inhibitors suppress the growth of neuroblastoma xenografts

**Fig. 5:**
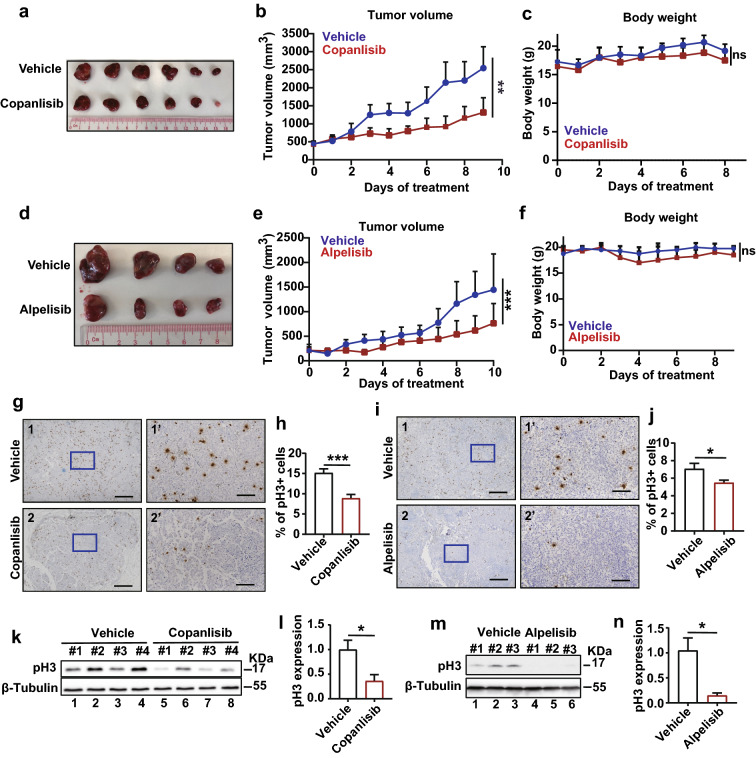
Copanlisib and alpelisib suppress neuroblastoma xenografts growth in nude mice. **a–c** KELLY cells were inoculated to nude mice subcutaneously to establish tumor xenograft. Tumor-bearing mice were treated with either vehicle (PBS) or copanlisib dihydrochloride every 2 days (intraperitoneal injection, 6 mg/kg, 9 days). The tumor volume (**b**) and body weight (**c**) of each mouse were recorded. Isolated tumors were pictured (**a**) on the last day. **d–f** KELLY cells were subcutaneously injected into nude mice. Tumor-bearing mice were then treated with vehicle (5% DMSO, 40% PEG300, 5% Tween-80, 50% PBS) or alpelisib (daily, oral gavage, 30 mg/kg, 10 days). **g–j** Tumor xenografts were processed to paraffin embedding and sectioning. pH3 was detected by immunohistochemistry (n = 4). Low magnification (**g1-2**, **i1-2**, scale bar = 500 µm) and higher magnification (**g1’-2’**, **i1’-2’**, scale bar = 100 µm) views were shown. pH3 positive cells were counted with Fiji (**h**, **j**). **k–n** Total pH3 levels in each tumor sample were detected by western blot. Quantification of the results was performed with Image J (**l**, **n**). Statistical significance was analyzed with Wilcoxon Signed Rank Test. *p < 0.05; **p < 0.01; ***p < 0.001; ns, not significant

To evaluate the efficacy of the PI3K inhibitors in the treatment of neuroblastoma in vivo, neuroblastoma xenografts in BALB/c nude mice were established and treated with copanlisib or alpelisib. As expected, copanlisib significantly suppressed the growth of neuroblastoma xenografts (Fig. 5a, b) with no differences in the body weight during the treatment period (Fig. 5c), suggesting the mild toxicity of the inhibitor. Similar tumor growth inhibition could be detected when treating the mice with alpelisib (Fig. 5d–f). Furthermore, tumor cells stained positive with pH3 in copanlisib-treated or alpelisib-treated tumor samples were reduced by 41% and 22% respectively, compared with those in vehicle-treated counterparts (Fig. 5g–j), indicating the critical role of p110α in the tumor formation of neuroblastoma. In line with this observation, copanlisib and alpelisib treatment also decreased pH3 levels in the neuroblastoma xenografts (Fig. 5k–n).

### p110α inhibition enhances the suppressive effects of ALK inhibitors on neuroblastoma cells

**Fig. 6:**
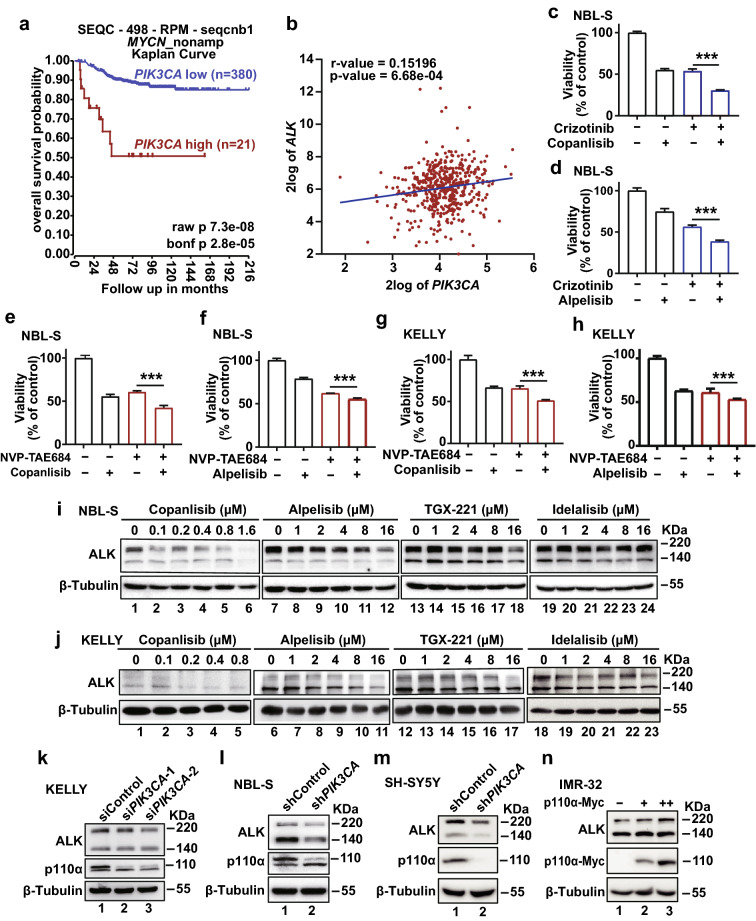
p110α inhibition decreases ALK in neuroblastoma cells. **a** Kaplan–Meier analysis of overall survival probability in neuroblastoma patients, separated by the expression of *PIK3CA* (SEQC (seqcnb1) dataset). The cut-off value was defined by Kaplan scan in the R2 platform (“Materials and methods” section)*.* **b** Expression correlation between *PIK3CA* and *ALK* in tumor samples in SEQC (seqcnb1) dataset. **c-h**, NBL-S and KELLY cells were treated with p110α inhibitors (copanlisib and alpelisib) and ALK inhibitors (crizotinib and NVP-TAE-684) alone or in combination. After 72 h, cell viabilities were detected by CellTiter-Glo® Luminescent Cell Viability Assay. Data were presented as mean ± SEM and analyzed with the Student’s *t*-test for statistical significance; *p < 0.05; **p < 0.01; ***p < 0.001. **i**, **j** ALK levels in NBL-S and KELLY cells were determined by western blot after treating with different PI3K inhibitors at indicated doses. **k–m** ALK levels were determined by western blot after knockdown of p110α with either shRNA or siRNA (50 nM, 48 h). β-Tubulin was used as the loading control. **n** ALK levels after p110α overexpression in IMR-32 cells were detected by western blot. β-Tubulin served as the loading control

We next studied the underlying mechanisms of the growth suppression of neuroblastoma induced by these p110α inhibitors. *MYCN* amplification is a genetic cause of neuroblastoma, and it was reported that inhibition of the PI3K-AKT-mTOR signaling pathway could inhibit neuroblastoma growth by destabilizing MYCN [19]. We also confirmed that *MYCN*-amplified cell lines, such as KELLY, LA1-5s, and BE(2)C, were relatively sensitive to PI3K inhibitors (Fig. 2b–d). On the other hand, according to the survival analysis from the R2 database, in patients without *MYCN* amplification, high expression of *PIK3CA* was associated with poor outcomes (Fig. 6a). Non-*MYCN* amplified neuroblastoma cells (NBL-S, SH-SY5Y, and SK-N-AS) also responded to the presence of p110α inhibitors (Fig. 2b–d). These data suggested the existence of other oncogenic targets mediated by the PI3K signaling pathway in neuroblastoma.

ALK is often overexpressed or constitutively activated by gain-of-function mutations in neuroblastoma, and ALK inhibitors have been tested in clinical trial for neuroblastoma treatment. We found that expression of *ALK* and *PIK3CA* were positively correlated in neuroblastoma samples (Fig. 6b). To test whether the combination of ALK and p110α inhibitors could improve the neuroblastoma treatment, we performed cell viability assays in vitro. In our assays, the *ALK*^*wt*^ carrying cell line NBL-S was sensitive to the first-generation ALK inhibitor (crizotinib) (IC50 value = 0.7934 μM) and the second-generation ALK inhibitor (NVP-TAE684) (IC50 value = 0.4996 μM) (Additional file 1: Fig. S7a, b). KELLY cells harbor *ALK*^*F1174L*^ mutation (a crizotinib-resistant mutation), but they were sensitive to NVP-TAE684 (IC50 value = 0.09008 μM) (Additional file 1: Fig. S7c). In the combinatory tests, the inhibitory effects of ALK inhibitors (crizotinib and NVP-TAE684) on the growth of NBL-S cells were significantly enhanced by either alpelisib or copanlisib (Fig. 6c–f). Similarly, NVP-TAE684 also synergized with either copanlisib or alpelisib to suppress the growth of KELLY cells (Fig. 6g, h). These results provide a strong rationale for testing the combinational use of ALK and p110α inhibitors to treat neuroblastoma in the clinic.

### Inhibition of PI3K activity reduced ALK by protein destabilization

PI3K-mediated signaling is one of the downstream signaling cascades upon ALK activation. We next examined whether ALK is regulated by the PI3K signaling reciprocally. As revealed in our experiments, p110α inhibitors suppressed PI3K signaling pathway and inhibited ALK expression in SH-SY5Y cells (Additional file 1: Fig. S7d, e). In a more detailed examination in NBL-S cells, ALK expression was decreased in a dose-dependent manner when treated with inhibitors of p110α but not p110β or p110δ (Fig. 6i). Similar results were obtained in BE(2)C cells expressing wild-type ALK (Additional file 1: Fig. S7f) and KELLY cells expressing oncogenic ALK mutant (Fig. 6j). Knockdown of p110α in neuroblastoma cells could also reduce ALK at the protein level (Fig. 6k–m), while overexpression of p110α upregulated ALK (Fig. 6n).

**Fig. 7:**
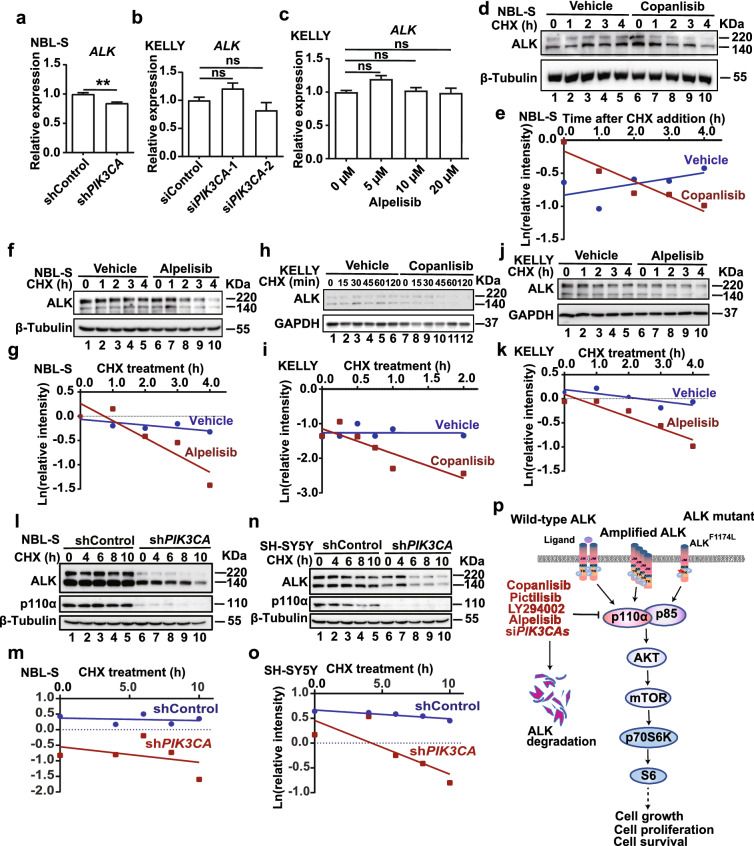
p110α inhibition destabilizes ALK protein in neuroblastoma cells. **a**, **b** mRNA levels of *ALK* were detected by qPCR after p110α inhibition by shRNA or siRNA (50 nM, 48 h) in NBL-S cells (**a**) and KELLY cells (**b**), respectively. **c** KELLY cells were treated with different doses of alpelisib for 24 h and *ALK* mRNA was analyzed by qPCR. *RPL19* expression was used as the internal control in **a-c**. Data are presented as mean ± SEM and analyzed with the Student’s *t*-test for statistical significance; *p < 0.05; **p < 0.01; ***p < 0.001; ns, not significant. **d–k** NBL-S and KELLY cells were incubated with the vehicle, copanlisib (0.5 µM), or alpelisib (2 µM) for 24 h, and analyzed by western blot at different time points after cycloheximide (100 µg/ml) treatment. **l**–**o** Cells with p110α knockdown were treated with cycloheximide (100 µg/ml) for different time, and ALK protein levels in these samples were detected by western blot to study ALK degradation. Quantification for each experiment was performed by Image J. The regression line for ALK degradation was then generated by GraphPad Prism. **p** The proposed mechanism of p110α inhibition induced tumor suppression in neuroblastoma

However, compared with the protein reduction in p110α-deficient NBL-S cells, the mRNA levels of *ALK* were only reduced in a mild manner (Fig. 7a and Additional file 1: Fig. S8a). In KELLY cells, knockdown of *PIK3CA* expression did not affect the *ALK* mRNA expression (Fig. 7b and Additional file 1: Fig. S8b). Moreover, inhibition of p110α activity using alpelisib could not downregulate *ALK* mRNA in KELLY cells (Fig. 7c). Consistent with previous results, the pan-PI3K inhibitor LY294002 also downregulated the ALK protein dose-dependently (Additional file 1: Fig. S9a), but no apparent decrease occurred at the mRNA levels upon the treatment of LY294002 (Additional file 1: Fig. S9b). Therefore, p110α likely affects the protein degradation rather than mRNA transcription of *ALK*. In cycloheximide (CHX) chase analysis of ALK degradation, the presence of copanlisib and alpelisib shortened the half-life of ALK protein significantly in NBL-S cells (Fig. 7d–g). In KELLY cells, the degradation of the ALK^F1174L^ mutant was also enhanced upon the treatment of copanlisib and alpelisib (Fig. 7h–k). In line with these observations, ALK protein was degraded faster in the presence of LY294002 (Additional file 1: Fig. S9c, d). Consistently, the shortened half-life of ALK protein was also detected in both ALK^wt^ (NBL-S) and ALK^F1174L^ (SH-SY5Y) cells when p110α expression was inhibited by shRNA (Fig. 7l–o). Thus, inhibition of PI3K, at least in part, decreases ALK protein stability, and negatively attenuates the inputs of ALK oncogenic signaling (Fig. 7p).

## Discussion

The regulatory role of the PI3K signaling pathway towards MYCN has been established in neuroblastoma previously, suggesting the PI3K signaling pathway as potential targets for neuroblastoma treatment [19]. In this study, we provided additional mechanisms underlying the role of specific catalytic PI3K isoforms in neuroblastoma biology with p110α highlighted. Our data demonstrated that p110α is a promising therapeutic target and represents the most important catalytic isoform among class IA PI3Ks in neuroblastoma. Notably, copanlisib and alpelisib are front-line used drugs in clinic to treat relapsed follicular lymphoma and metastatic breast cancer, respectively [21, 22]. These two compounds have great potentials for combination treatment of neuroblastoma patients in the clinic. Other PI3K inhibitors with high potential in clinical application and having p110α as a target, such as buparlisib and serabelisib, are worthy of testing for neuroblastoma treatment [23, 24].

We also identified ALK as a downstream effector mediated by p110α inhibition. The application of several ALK inhibitors in treating high-risk neuroblastoma patients is under development in clinical trials [25]. However, therapeutic resistance has been reported with all ALK inhibitors in neuroblastoma and other tested cancers. Therefore, it is highly significant to develop appropriate combination regimens and to elucidate in-depth mechanisms of ALK in carcinogenesis. Encouragingly, we showed that p110α inhibitors promote the suppressive effects of ALK inhibitors in neuroblastoma, so the presence of PI3K inhibitors may help to antagonize the drug resistance of neuroblastoma. Regardless of the importance of ALK in neuroblastoma, the cellular functions of full-length ALK in both physiological and oncogenic contexts are largely unknown. Our findings suggest a mechanism that suppression of the PI3K signaling pathway reduces the ALK stability. Since the PI3K signaling pathway is a downstream cascade of the ALK activation, they may form a positive feedback loop in the oncogenesis of neuroblastoma (Fig. 7p). However, the underlying mechanism as to how PI3K signaling mediated ALK stability is still poorly understood. Bruton’s tyrosine kinase (BTK) was reported to increase the ALK stability by reducing the ALK ubiquitination [26]. It is worthwhile to investigate the correlation of BTK and PI3K on ALK protein stability in the future.

## Conclusions

Our findings suggest that neuroblastoma was p110α-dependent rather than relying on p110β or p110δ in the activation of the PI3K signaling pathway, thus providing detailed information for the future intervention of neuroblastoma by targeting the PI3K signaling pathway. We also highlighted the U.S. Food and Drug Administration-approved agents, copanlisib, and alpelisib, as potential drugs for treating neuroblastoma. Mechanistically, we demonstrated that the ALK degradation is induced by the p110α inhibition. Our data suggest that the combinational application of ALK and p110α inhibitors is worth testing to treat ALK-positive neuroblastoma.

## Materials and methods

### Cell culture

Neuroblastoma cell lines with *MYCN* amplification (LA1-5s, IMR-32, BE(2)C, LAN-5, and KELLY) and with non-*MYCN* amplification (SH-SY5Y, NBL-S, and SK-N-AS) were used in this study [26–31]. IMR-32 and SH-SY5Y were purchased from DSMZ (Braunschweig, Germany). BE(2)C was from ATCC (Manassas, Virginia, USA), and KELLY was from Sigma (Burlington, Massachusetts, USA). Other cell lines are from our lab stock. LA1-5 s, SK-N-AS, and BE(2)C cells were grown in DMEM with 10% fetal bovine serum (FBS). SH-SY5Y cells were cultured in DMEM with 12.5% FBS. KELLY was grown in RPMI with 10% FBS. LAN-5 and IMR-32 were cultured in RPMI with 10% FBS.

### Inhibitors and antibodies

The PI3K inhibitor copanlisib, TGX-221, alpelisib, idelalisib, and LY294002 were purchased from MedChemExpress (Princeton, New Jersey, USA). The PI3K inhibitor pictilisib, the ALK inhibitor crizotinib and NVP-TAE684 were obtained from ADOOQ (Irvine, California, USA). The antibodies against ALK, phospho-ERK1/2, Akt1/2, N-Myc, GAPDH, and beta-actin were purchased from Santa Cruz Biotechnology (Santa Cruz, California, USA). Antibodies targeting Phospho-Akt (Ser473), Phospho-S6 Ribosomal Protein (Ser235/236), S6 Ribosomal Protein, and anti-Phospho-Histone H3 (Ser10) were purchased from Cell Signaling Technology (Danvers, Massachusetts, USA). Other antibodies used in this study included anti-MAPK1/2 (Milipore, NG1946), anti-β-Tubulin antibody (Abcam, Ab6046).

### Knockdown and overexpression of p110α

*PIK3CA* expression was knocked down by small interfering RNA (siRNA) in neuroblastoma cells. The sequences of siRNAs were provided in Additional file 1: Table S1. The siRNAs were transfected into neuroblastoma cells using Lipofectamine 3000 Reagent (Invitrogen, 11,668,019), and cells were collected 48 h after transfection. To establish the stable *PIK3CA* knockdown cell line, constructs (shControl and sh*PIK3CA*) were generated by inserting the modified sequences of siControl and si*PIK3CA*-1 into pLKO.1. Virus packaging and puromycin selection were then performed according to the methods described previously [32]. To overexpress p110α in neuroblastoma cells, protein coding sequence of *PIK3CA* was inserted into pCS2 + MT (in frame with Myc tag) and transfected into neuroblastoma cells with Lipofectamine 3000 reagent. Cells were harvested and analyzed 48 h after transfection. Western blot and real-time PCR were employed to assess the levels of p110α.

### Quantitative real-time PCR

Total RNA was extracted using TRI Reagent® Solution (Ambion™) according to the standard protocol and quantified by a NanoDrop ND-2000 (NanoDrop Technologies). cDNA was reverse transcribed from total RNA using the PrimeScript™ RT reagent Kit (TaKaRa, RR037A). TB Green Premix Ex Taq (Tli RNase H Plus) (TaKaRa, RR420A) was used for quantitative real-time PCR (qPCR) analysis. The target genes were amplified in an ABI Prism 7500 Sequence Detection System (Applied Biosystems). The primer sequences were listed in Additional file 1: Table S2.

### Western blot

Cells were washed once in ice-cold PBS and lysed in the lysis buffer containing 1% Triton X-100, 25 mM Tris (pH 7.5), 150 mM NaCl, 5 mM EDTA, 10% glycerol, aprotinin (1 µg/ml), leupeptin (1 µg/ml), pepstatin A (1 µg/ml), sodium orthovanadate (1 mM), and 1 mM phenylmethylsulfonyl fluoride. The lysates were mixed with SDS sample buffer and separated by SDS–polyacrylamide gel, followed by electrotransfer to Immobilon P membranes (Millipore). Membranes were blocked in TBST (0.1% Tween-20 in TBS) with 5% nonfat milk or 1% BSA for 1 h at room temperature and incubated with primary antibodies overnight at 4 °C. After washing with TBST, membranes were incubated with horseradish peroxidase-conjugated secondary antibody for 2 h at room temperature and then washed with TBST. Chemiluminescent signals were developed with Millipore ECL reagent (Millipore, WBLUF0500) and captured by SYNGENE G: box. Image J software was used to quantify the signal intensity of the target bands [33].

### Cell viability assay

Cells were first seeded in 96-well plates. At the indicated time, CellTiter-Glo® Luminescent Cell Viability Assay was performed according to the manufacturer’s instruction (Promega, G7571). Puromycin (10 μg/ml) was applied for non-survival controls. To determine the IC50 values of inhibitors, vehicle (maxi) and non-survival control (min) were included. Before illustrating the cell viability curves, all values tested for samples were normalized with the formula: (Value_sample_ -Mean_min_)/(Mean_maxi_- Mean_min_)*100%. IC50 values were then determined after fitting curves using GraphPad Prism (https://www.graphpad.com/support/faqid/1566/).

To illustrate the cell growth curves, 10 µl 3-(4,5-Dimethylthiazol-2-yl)-2,5-diphenyltetrazolium bromide (MTT) (5 μg/ml) (Sigma, M5655) was added to each well followed by incubation for 4 h. The absorbance was measured at A570 nm and A630 nm. The values were calculated by A_570 nm_-A_630 nm_, then normalized to that in control (Day 1). All samples were assayed with five repeats, and each experiment was performed three times.

### Colony formation assay

Cells were seeded in 6-well dishes (5000 cells per well) and treated with indicated inhibitors. Seven to eleven days later, the cells were fixed and stained with 0.5% crystal violet (Sigma, C0775) in 25% methanol. The number of colonies was counted using Image J [33].

### Cell cycle analysis

Propidium Iodide (PI) staining was used for the cell cycle analysis. After 24-h inhibitor treatment, cells were trypsinized and washed with PBS. They were further fixed with 70% ice-cold ethanol overnight and washed with PBS, followed by staining in freshly prepared nuclei staining buffer (0.1% Triton X-100 in PBS, 200 μg/ml RNase, and 20 g/ml PI) for 15 min at 37 °C. The cells were then analyzed by flow cytometry. Histograms were generated by Flow Jo to determine the percentage of cells in each phase (G_0_/G_1_, S, and G_2_/M).

### Tumor xenograft

KELLY cells (6 × 10^6^) in 100 µl of PBS supplemented with 50% of Matrigel (Corning, 354234) were subcutaneously injected into the right flank of nude mice (3–4 weeks old, female). Tumor-bearing mice were grouped randomly when tumors were visible. To test the efficacy of copanlisib, these mice were treated with either copanlisib dihydrochloride (6 mg/kg) or PBS through intraperitoneal injection every other day for 9 days (6 mice per group). To evaluate the efficacy of alpelisib, mice bearing tumors were treated with alpelisib (30 mg/kg) or its vehicle (5% DMSO, 40% PEG300, 5% Tween-80, 50% PBS) orally every day for 10 days. The tumor volume and body weight of each mouse were monitored every day. Tumor volume was calculated by length × width^2^/2. The length stands for the long side, and the width stands for the short side.

### Immunohistochemistry

Xenograft tumors were fixed and embedded in paraffin. Tumor sections were further processed for deparaffinization and rehydration. After antigen retrieval and blocking, tumor sections were incubated with the Phospho-Histone H3 (pH3) antibody (Cell Signaling Technology, 3377S) at 4 ℃ overnight, then incubated with the HRP-conjugated secondary antibody (Dako, p0448). Tumor sections were further incubated with DAB solution for chromogenic detection and counterstained with hematoxylin. Stained samples were then visualized and imaged under a bright light microscope. The quantification of the pH3 positive cells was performed with Fiji [34].

### Gene expression analysis in tumor samples and survival analysis

Kaplan–Meier analysis was applied to analyze the survival probability of patients with high and low expression of *PIK3CA*, *PIK3CB*, and *PIK3CD* in the R2: Genomics Analysis and Visualization Platform (http://r2.amc.nl) with the default setting. Briefly, the cutoff was set by the scan function with the minimal group number of 8. The Kaplan scanner sorts the samples first according to the expression levels. Using every increasing expression value as a cutoff, the scanner then separates samples into two groups and performs the logrank test. The best *p* value and the corresponding cutoff will be used, and a corrected *p* value from multiple testing (Bonferroni correction) is also included. Megasampler tool in the R2: Genomics Analysis and Visualization Platform was used to evaluate the expression levels of *PIK3CA* in normal and neuroblastoma datasets (Affymetrix Human Genome U133 Plus 2.0 Array, normalized with MAS5.0). For detailed expression analysis of *PIK3CA* in neuroblastoma across The International Neuroblastoma Staging System (INSS) stages or different risk groups, SEQC (seqcnb1) dataset was analyzed by “view a gene in groups” and tracked accordingly.

### Statistical analysis

Statistical analysis was performed using GraphPad Prism 5.0. The results were presented as mean ± standard error (SD) or mean ± standard error of mean (SEM). The Student’s *t*-test or Wilcoxon Signed Rank Test was used for the hypothesis testing. A *p*-value less than 0.05 was considered significant. The statistical significance levels were set as * *p* < 0.05, ** *p* < 0.01, and *** *p* < 0.001, respectively.

## Supplementary Information

**Additional file 1: Fig. S1–S9.** Additional figures, **Table S1.** siRNA sequences for knockdown assays, and **Table S2.** primers for qPCR.

## Data Availability

The datasets analyzed in this study are available from the R2: Genomics Analysis and Visualization Platform (http://r2.amc.nl). Materials and experimental data are achievable from the corresponding authors upon request.

## Abbreviations

ALK: Anaplastic lymphoma kinase
BTK: Bruton’s tyrosine kinase
CHX: Cycloheximide
FBS: Fetal bovine serum
INSS: The International Neuroblastoma Staging System
MTT: 3-(4,5-Dimethylthiazol-2-yl)-2,5-diphenyltetrazolium bromide
MYCN: N-MYC proto-oncogene protein
pH3: Phospho-Histone H3
PI: Propidium Iodide
PI3K: PI3 Kinase
qPCR: Quantitative real-time PCR
SD: Standard error
SEM: Standard error of mean
siRNA: Small interfering RNAs
shRNA: Short hairpin RNA
