# Supplementary material for: Inhibition of PI3 kinase isoform p110α suppresses neuroblastoma growth and induces the reduction of Anaplastic Lymphoma Kinase
Source: Cell Biosci. 2022 Dec 30;12:210. doi: 10.1186/s13578-022-00946-9 (PMC9801621; doi:10.1186/s13578-022-00946-9)
Supplement: Supplementary file 1 — Additional file 1: Fig. S1–S9. Additional figures, Table S1. siRNA sequences for knockdown assays, and Table S2. primers for qPCR. [file 13578_2022_946_MOESM1_ESM.pdf]

Additional Materials

This PDF file includes:

Fig. S1 to S9

Table S1 siRNA sequences for knockdown assays

Table S2 Primers for q-PCR

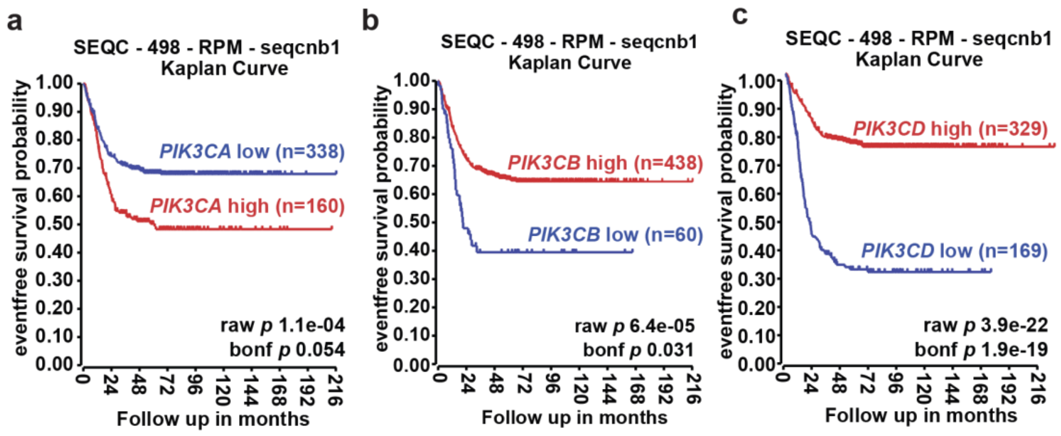

Fig. S1 High expression of *PIK3CA* is correlated with lower event-free survival probability in neuroblastoma patients. Kaplan-Meier analysis of event-free survival probability in neuroblastoma patients, separated by *PIK3CA* (**a**), *PIK3CB* (**b**), and *PIK3CD* (**c**) expression. Cut-off values were defined by Kaplan Scan in R2 platform. SEQC (seqcnb1) dataset was analyzed to generate this figure.

a

Characteristics of the Cell Lines

Cell line	Genetic Characteristics	
	MYCN Amplification	*ALK* Mutation
SH-SY5Y	No	F1174 L
NBL-S	No	wt
SK-N-AS*	No	wt
LA1-5s	Yes	no *ALK* expression
IMR-32	Yes	wt
BE(2)C	Yes	wt
LAN-5	Yes	R1275Q
KELLY	Yes	F1174 L

Note: * SK-N-AS is harboring *NRAS* mutation: Q61K.

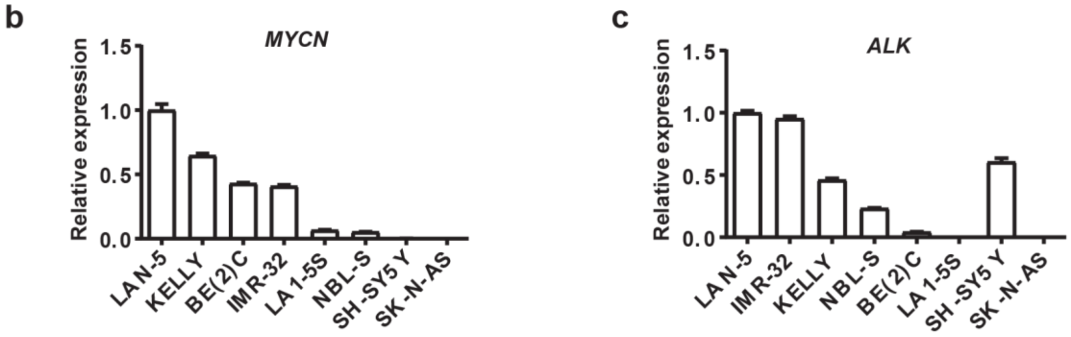

Fig. S2 Expression of *MYCN* and *ALK* in different neuroblastoma cell lines. **a**, genetic alterations of *MYCN* and *ALK* in the indicated neuroblastoma cell lines. **b-c**, qPCR analysis of *MYCN* and *ALK*. *RPL19* served as the endogenous control.

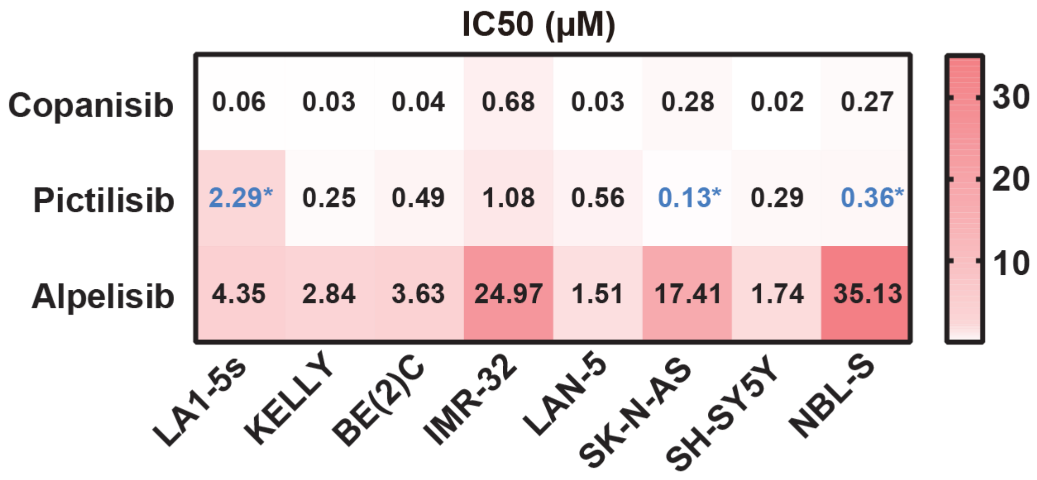

Fig. S3 IC50 values of different p110α-targeted inhibitors in neuroblastoma cell lines in cell viability assays. Neuroblastoma cells were treated with either copanlisib, pictilisib, or alpelisib at different doses for 72 hours. Cell viability was then evaluated by CellTiter-Glo® Luminescent Cell Viability Assay. IC50 values were determined based on the cell viability curves (Materials and Methods). Pictilisib treatment failed to reach 50% inhibition on cell viability in LA1-5s, SK-N-AS, and NBL-S cells due to its low solubility. "*" indicates inaccurate IC50s due to bad initial values when fitting the viability curve.

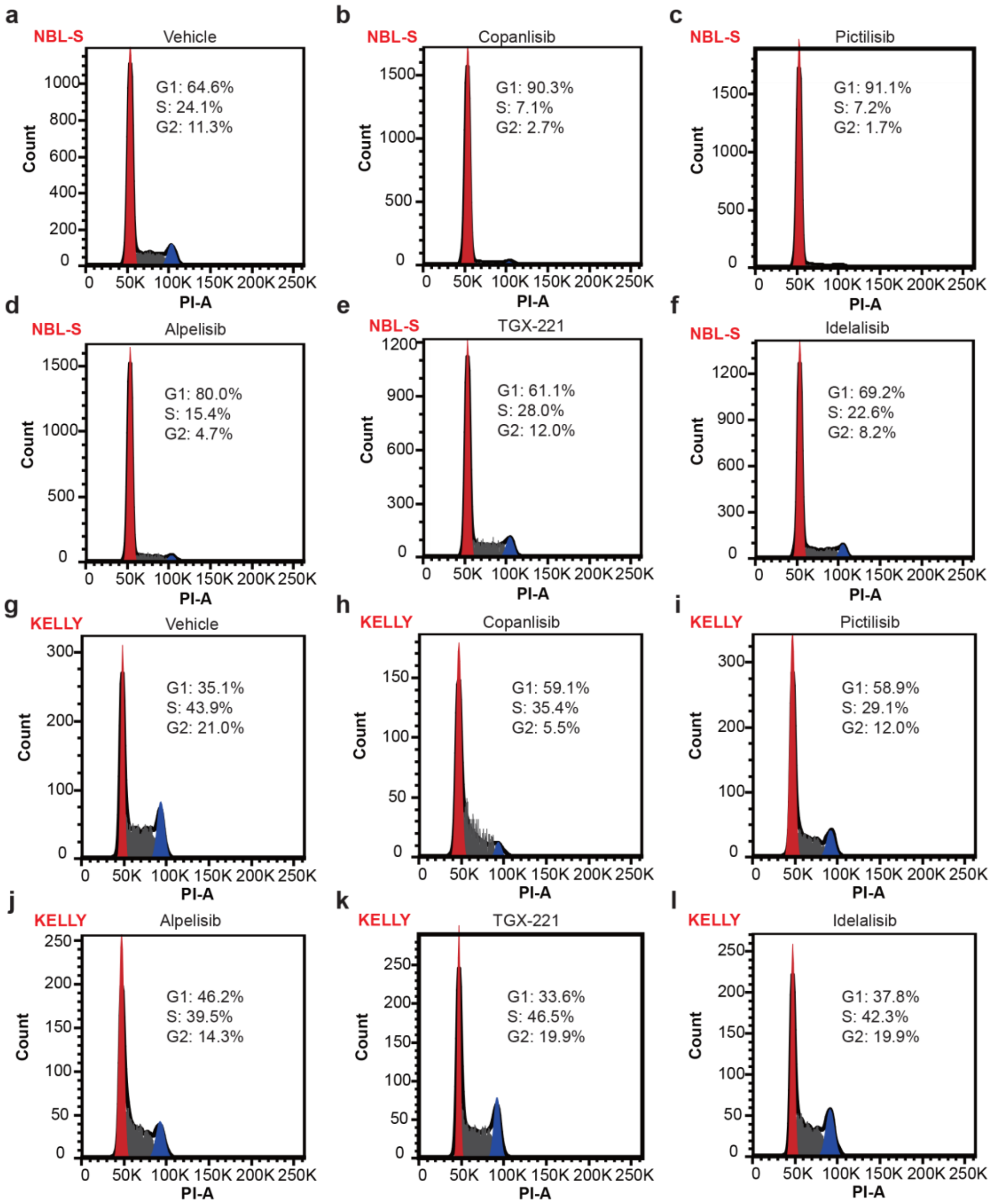

Fig. S4 p110α inhibition induces G_0/G_1 cell cycle arrest of neuroblastoma cells. NBL-S and KELLY cells were treated with different PI3K inhibitors for 24 hours (1 µM) and fixed for propidium iodide staining. DNA content was then profiled by flow cytometry analysis. Cell cycle distribution was further determined by Flow Jo software.

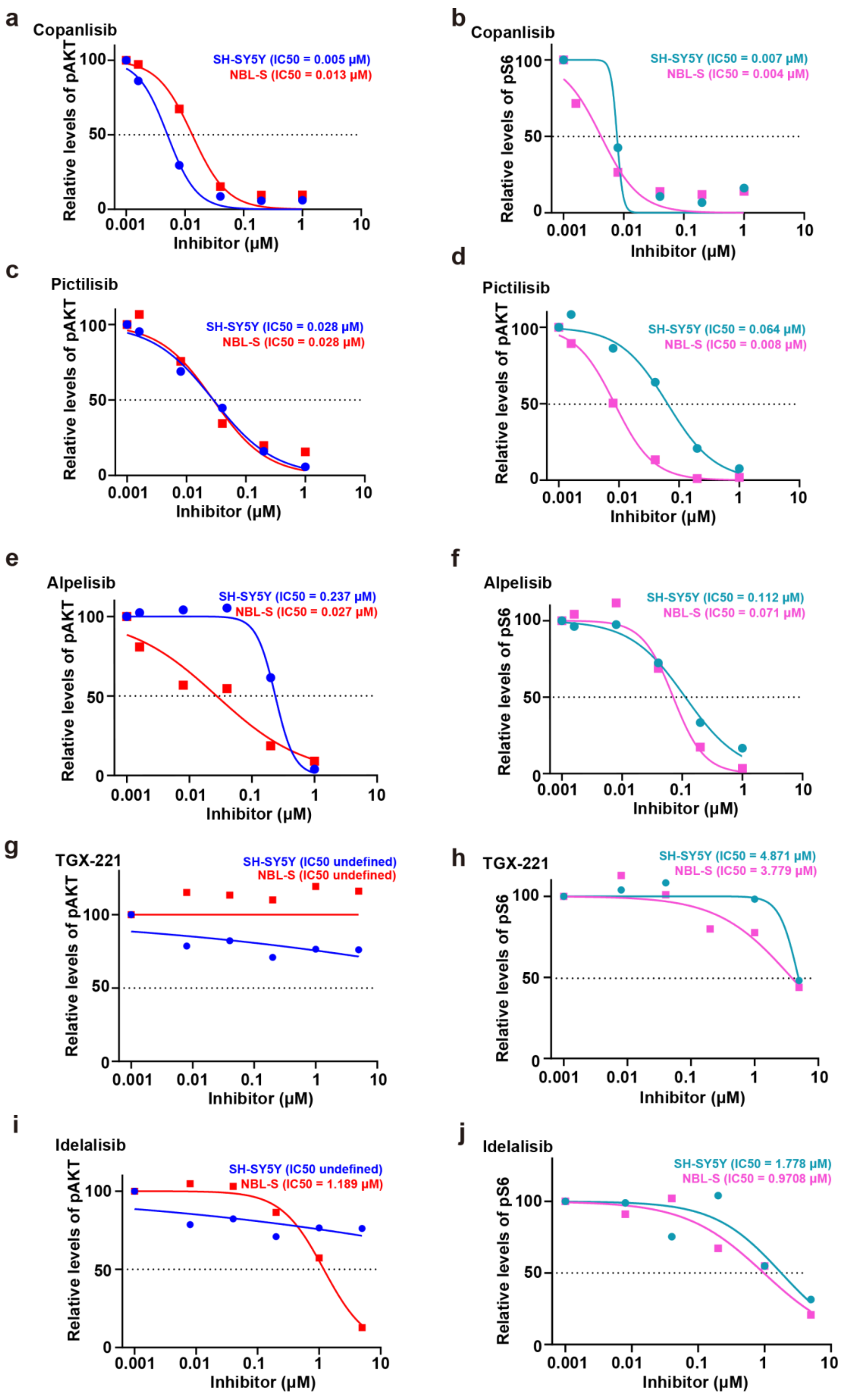

Fig. S5 p110α inhibitors suppress the PI3K signaling pathway. Inhibition curves of

pAKT and pS6 were generated using the quantification data from the western blot analysis after PI3K inhibitors treatment (2 hours). The signal intensity of each band was quantified with ImageJ. The intensity of pAKT and pS6 were then normalized to AKT and S6, respectively. The normalized data were inputted into GraphPad Prism to draw the curves. IC50 values were then determined according to the nonlinear regression curve fit results.

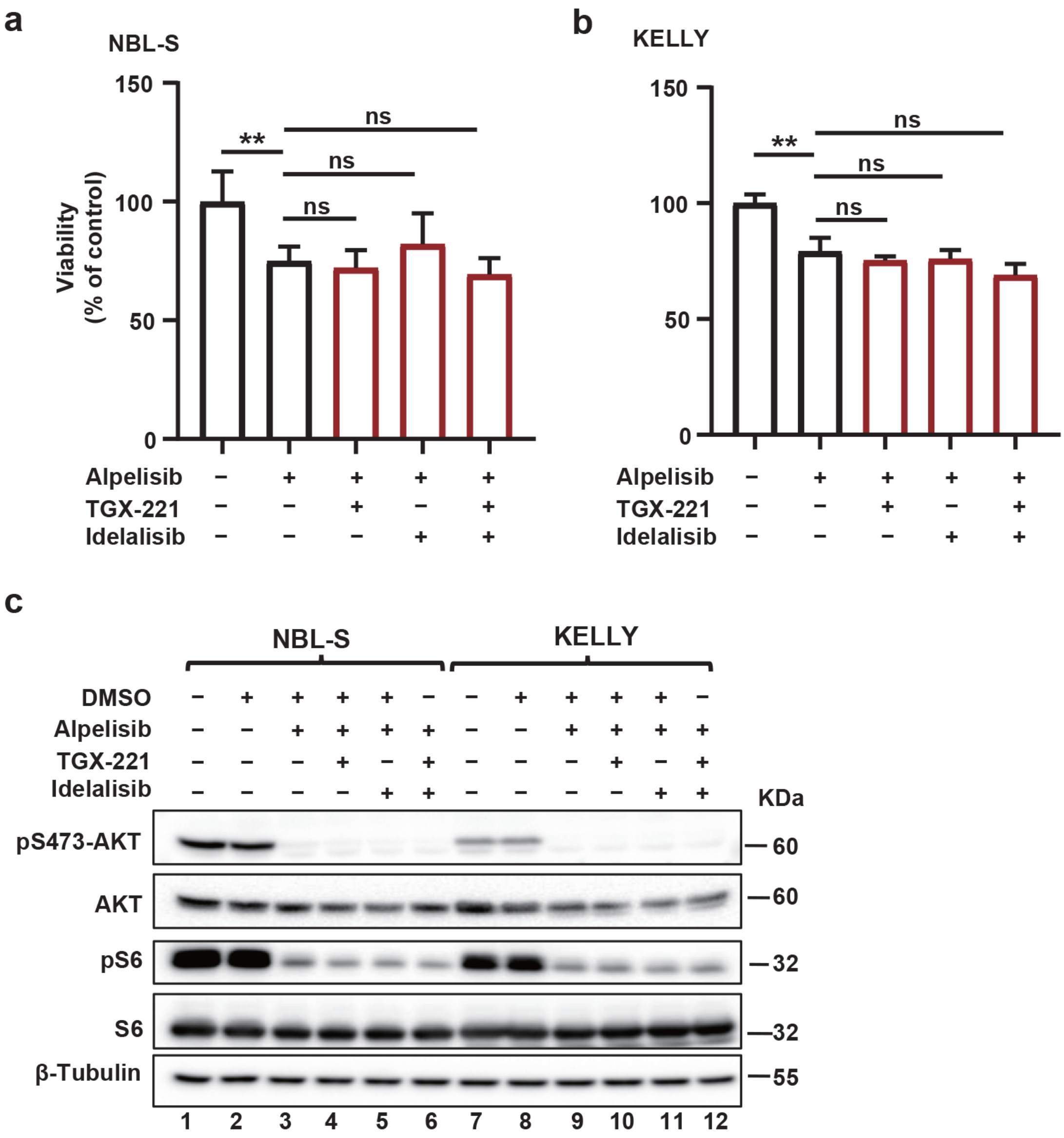

Fig. S6 Additions of TGX-221 and idelalisib do not enhance the suppressive effects of alpelisib on neuroblastoma cells. **a-b**, NBL-S, and KELLY cells were treated with alpelisib, TGX-221, and idelalisib as mono treatment or in combination (2 μM). Cell viability was evaluated after 48 hours with MTT assays. Data are presented as mean ± SEM, and statistical analysis was performed with the Student's *t*-test; **, $p < 0.01$; ns, not significant. **c**, NBL-S and KELLY cells were incubated with DMSO, alpelisib, TGX-221, and idelalisib as indicated for 18 hours. Cell lysates from the treated cells were further analyzed by western blot with indicated antibodies.

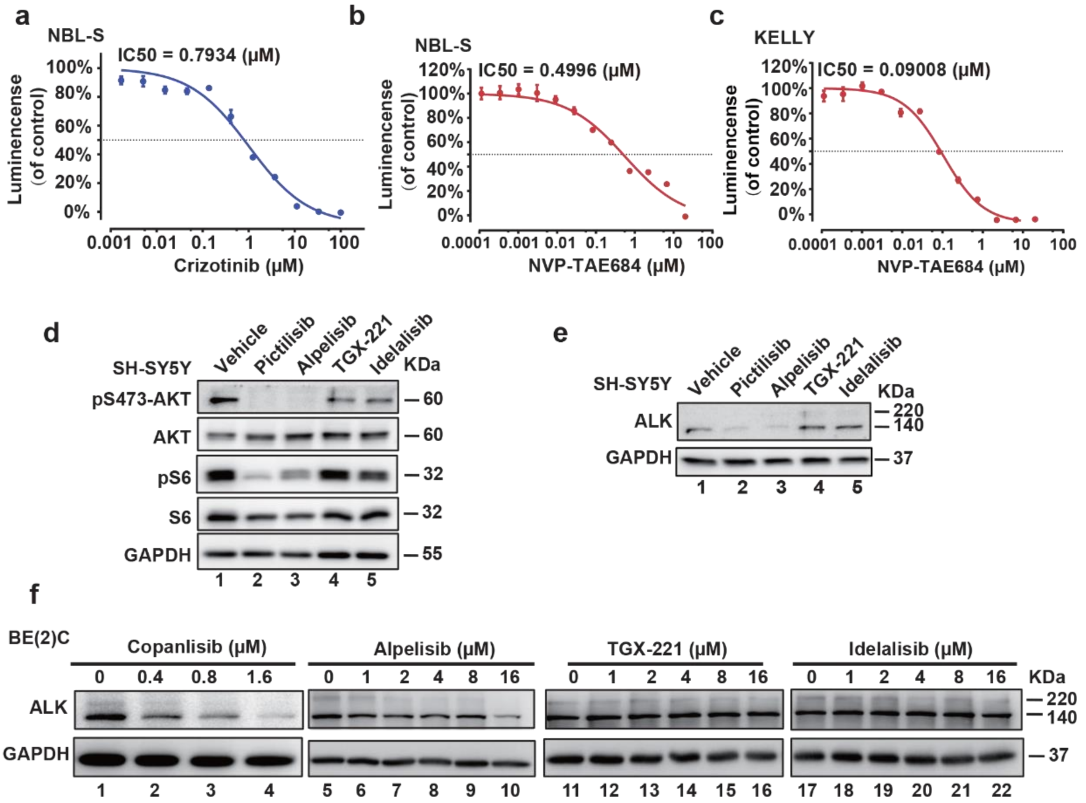

Fig. S7 PI3K inhibition decreases ALK in neuroblastoma. **a-c**, cell viability inhibition curves of NBL-S (a-b) and KELLY cells treated by ALK inhibitors (c). Cells were seeded in 96-well plates and treated with indicated inhibitors for 72 hours. At the endpoint, cell viability was determined by CellTiter-Glo® Luminescent Cell Viability Assay. Inhibition curves and IC50s were defined by GraphPad Prism (Materials and Methods). **d-e**, SH-SY5Y cells were treated with PI3K inhibitors (2 μM) as indicated for 24 hours. The whole-cell lysis was analyzed with western blot to check the PI3K signaling pathway and ALK expression. **f**, BE(2)C cells were treated with indicated inhibitors for 18 hours (2 μM). The cells were then harvested for western blot. Antibodies targeting ALK and GAPDH were used to determine their expression in different groups.

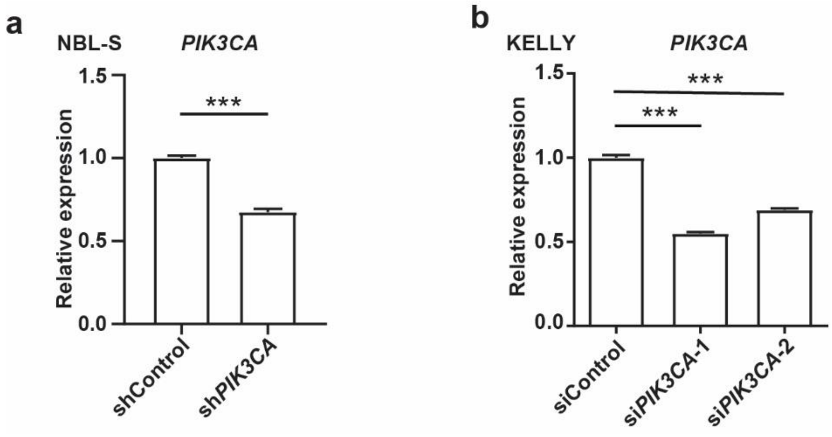

Fig. S8 *PIK3CA* expression was attenuated by siRNA and shRNA in neuroblastoma cells. shRNAs and siRNAs were designed to suppress p110α expression or serve as a control in neuroblastoma cells. mRNA levels of *PIK3CA* were validated with qPCR. *RPL19* was used as the internal standard control. Data are presented as mean ± SEM, and statistical analysis was performed with the Student's *t*-test; ***, $p < 0.001$.

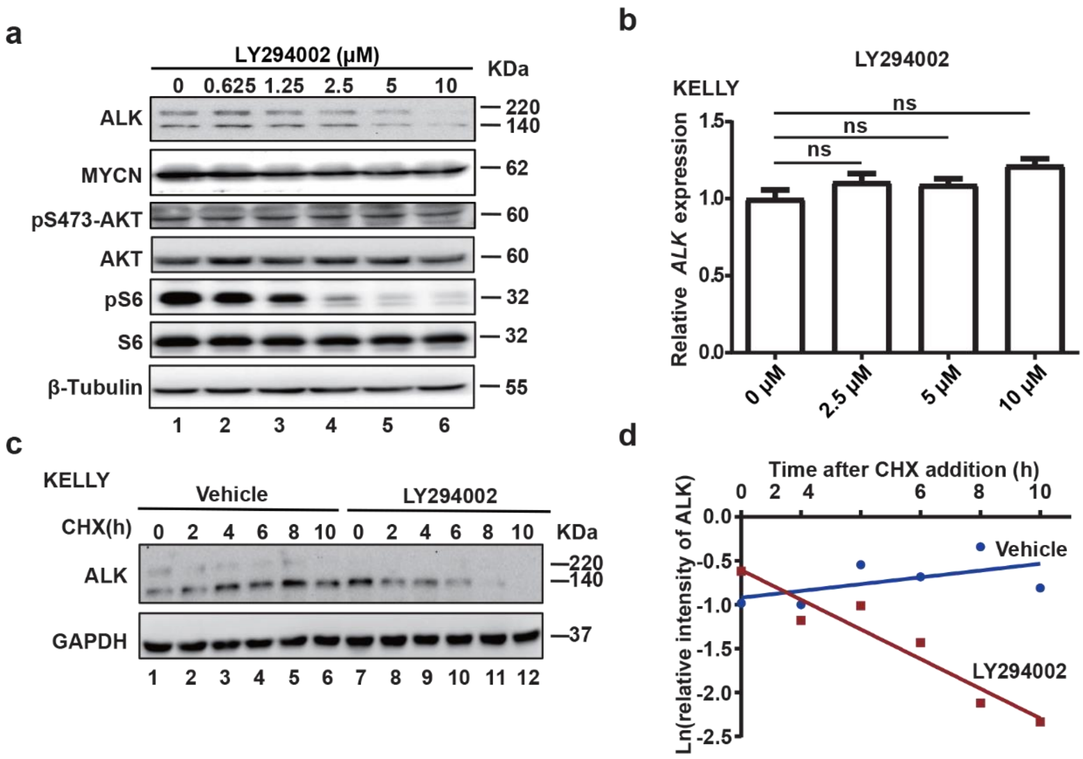

Fig. S9 LY294002 reduces ALK stability. **a-b**, KELLY cells were treated with LY294002 at different doses for 18 hours. The cells were then harvested for western blot (**a**) or qPCR analysis (**b**). *RPL19* was employed as the internal standard control for qPCR analysis. **c-d**, KELLY cells were treated with 5 μM of LY294002 for 12 hours and collected at the indicated time after incubating with CHX (100 μg/ml). Western blot was then performed to determine the ALK protein levels (**c**). The degradation curve was illustrated according to quantified results from the western blot (**d**).

Table S1 siRNA sequences for knockdown assays

Name	Primer sequence
siControl	UUCUCCGAACGUGUCACGUTT<br>ACGUGACACGUUCGGAGAATT
si*PIK3CA*-1	CCGUGAGGCUACAUUAAUATT<br>UAUUAAUGUAGCCUCACGGAG
si*PIK3CA*-2	GCCAGUACCUCAUGGAUUAGAAGAU<br>AUCUUCUAAUCCAUGAGGUACUGGC

All siRNAs were purchased from genepharma.

Table S2 Primers for qPCR

Gene ID	Gene name	Primer sequence
238	ALK	TCAGTCCACTGGGCATCCTGTA<br>GCAGATGACCTTGTGGCTTTCAG
4613	MYCN	ACCACAAGGCCCTCAGTACCTC<br>TGACAGCCTTGGTGTTGGAGGA
6143	RPL19	ACATGGGCATAGGTAAGCGGAAG<br>TTCACCTTCAGGTACAGGCTGTG
